# Bimetallic ZIF-Derived Co/N-Codoped Porous Carbon Supported Ruthenium Catalysts for Highly Efficient Hydrogen Evolution Reaction

**DOI:** 10.3390/nano11051228

**Published:** 2021-05-06

**Authors:** Hui Qi, Xinglong Guan, Guangyu Lei, Mengyao Zhao, Hongwei He, Kai Li, Guoliang Zhang, Fengbao Zhang, Xiaobin Fan, Wenchao Peng, Yang Li

**Affiliations:** Lab of Advanced Nano-Structure and Transfer Process, Department of Chemical Engineering, Tianjin University, Tianjin 300354, China; qihui1101@tju.edu.cn (H.Q.); tjugxl@tju.edu.cn (X.G.); guangyulei@tju.edu.cn (G.L.); Zhaomy@tju.edu.cn (M.Z.); hehongwei@tju.edu.cn (H.H.); tju-likai@tju.edu.cn (K.L.); zhangguoliang@tju.edu.cn (G.Z.); fbzhang@tju.edu.cn (F.Z.); xiaobinfan@tju.edu.cn (X.F.); wenchao.peng@tju.edu.cn (W.P.)

**Keywords:** bimetallic ZIF, porous carbon, ruthenium, hydrogen evolution reaction

## Abstract

Exploring the economical, powerful, and durable electrocatalysts for hydrogen evolution reaction (HER) is highly required for practical application. Herein, nanoclusters-decorated ruthenium, cobalt nanoparticles, and nitrogen codoped porous carbon (Ru-pCo@NC) are prepared with bimetallic zeolite imidazole frameworks (ZnCo-ZIF) as the precursor. Thus, the prepared Ru-pCo@NC catalyst with a low Ru loading of 3.13 wt% exhibits impressive HER catalytic behavior in 1 M KOH, with an overpotential of only 30 mV at the current density of 10 mA cm^−2^, Tafel slope as low as 32.1 mV dec^−1^, and superior stability for long-time running with a commercial 20 wt% Pt/C. The excellent electrocatalytic properties are primarily by virtue of the highly specific surface area and porosity of carbon support, uniformly dispersed Ru active species, and rapid reaction kinetics of the interaction between Ru and O.

## 1. Introduction

Among the promising clean energy sources, hydrogen has attracted intense investigation for its high energy density and zero emission (no greenhouse gases are emitted) [1,2,3]. Electrocatalytic water splitting to generate ultrapurity and pollution-free hydrogen is an effective and sustainable approach in the industry [4,5]. As a critical component of water electrolysis technology, hydrogen evolution reaction (HER) at the cathode urgently demands highly efficient and durable catalysts for large-scale application [6]. Until now, platinum (Pt)-based catalysts are constantly at the leading edge of most HER electrocatalysts [7,8]. However, the widespread development and application of Pt-based catalysts are held up because of the insufficient on-earth storage and high cost of Pt. Moreover, there is also an issue linked to the stability of Pt-based electrocatalysts in strong alkaline and acidic electrolytes [9]. Hence, developing other low-precious metal catalysts for the substitution of Pt has attracted more and more interest in recent years [10,11,12]. Ruthenium (Ru) is known to be the cheapest Pt-group metal, which just costs ~4% of Pt, yet it possesses a resembling metal-hydrogen binding strength (M–H*) to Pt (~65 kcal mol^−1^) and a stronger corrosion resistance [13,14]. Currently, numerous Ru-based catalysts have been designed and have shown pleasant catalytic behaviors for HER. For example, Chang et al. prepared the pure RuP*_x_* catalysts via a simple and controllable heat-treatment method, which exhibited impressive catalytic performance for pH-universal HER [15]. Zong et al. reported the core-shelled Fe@Ru nanoparticles (4.8 wt% Ru) enclosed in N-doped carbon nanoframes, which presented an overpotential of 55 mV at 10 mA cm^−2^ (*η*_10_) and a Tafel slope of 77.3 mV dec^−1^ for alkaline HER [16].

Finding a suitable material as the support for active centers is also crucial to ensure the effective interactions between the different phases involved in most electrocatalytic reaction processes [17,18]. Recently, the preparation of metal/carbon-based porous materials using metal organic frameworks (MOFs) as precursors has emerged as a hot research issue [4,19,20]. Zeolite imidazole frameworks (ZIFs), as a new type of MOFs with zeolite-like 3D topological structure, are considered promising to derive nitrogen-rich carbon with highly specific surface area (SSA), abundant porosity, and good structural stability [21,22]. Among them, zinc (Zn)-based ZIF-8 can afford highly microporous nitrogen-doped carbons with large SSA and high porosity after thermal activation, whereas it cannot provide well-graphitized carbon, leading to low electrical conductivity [23]. On the other hand, cobalt (Co)-based ZIF-67 can offer enhanced electrical conductivity due to the catalytic graphitization by Co nanoparticles (NPs) but causes the loss of SSA and porosity [24]. Hence, if the two are combined, high SSA and conductivity can be achieved at the same time.

In the view of the above considerations, a highly active electrocatalyst by introducing Ru nanoclusters into the Co/N-codoped porous carbon born of a bimetallic ZIF (ZnCo-ZIF) is reported (denoted as Ru-pCo@NC) in this study. Benefiting from the good electrical conductivity, large SSA, uniformly dispersed active sites, and rapid kinetics induced by the interaction between Ru and O, Ru-pCo@NC with low Ru loading content (3.13 wt%) display impressing catalytic properties toward HER in 1 M KOH, with *η*_10_ of 30 mV, Tafel slope of 32.1 mV dec^−1^, and high durability, rivaling commercial 20 wt% Pt/C and outperforming most Ru-based electrocatalysts that have been studied (Table S1).

## 2. Materials and Methods

### 2.1. Materials

All chemicals are commercial and used without further purification. Zinc (II) nitrate hexahydrate (Zn(NO_3_)_2_·6H_2_O), cobalt (II) nitrate hexahydrate (Co(NO_3_)_2_·6H_2_O) and methanol (CH_4_O) were purchased from Kermel Chemical Reagent Co. Ltd. (Tianjin, China). 2-methylimidazole (C_4_H_6_N_2_) and commercial Pt/C (20 wt%) were purchased from Macklin Biochemical Co. Ltd. (Shanghai, China). Ruthenium (III) chloride (RuCl_3_) was purchased from Heowns Biochemical Technology Co. Ltd. (Tianjin, China). Nafion solution (5%) and potassium hydroxide (KOH) were purchased from Sigma-Aldrich Co. Ltd. (Shanghai, China).

### 2.2. Synthesis of ZIF Precursors

For the synthesis of ZIF-67, 0.02 mol Co(NO_3_)_2_·6H_2_O and 0.08 mol 2-methylimidazole (2-MeIm) were dissolved in 60 mL methanol (MeOH) to form two solutions, respectively. Then, the solution of 2-MeIm was added to the solution of Co(NO_3_)_2_·6H_2_O with vigorous stirring for 24 h at room temperature. The resulting purple product was collected by centrifugation, washed thoroughly with water and MeOH for several times, and finally dried in vacuum at 60 °C overnight.

ZIF-8 was formed by the similar method above except that the Co(NO_3_)_2_·6H_2_O was replaced by Zn(NO_3_)_2_·6H_2_O.

For the synthesis of ZnCo-ZIF, the mixture of 0.014 mol Zn(NO_3_)_2_·6H_2_O and 0.006 mol Co(NO_3_)_2_·6H_2_O were dissolved in 60 mL MeOH to form a mixture solution. Then, a solution of 0.08 mol 2-MeIm in 60 mL MeOH was added to the above mixture solution with vigorous stirring for 24 h at room temperature. The resulting light-purple product was collected by centrifugation, washed thoroughly with water and MeOH several times, and finally dried in vacuum at 60 °C overnight.

### 2.3. Synthesis of pCo@NC, NC, and Co@NC

For the preparation of pCo@NC, the as-synthesized ZnCo-ZIF nanocrystals were transferred into a corundum boat and placed in the tube furnace under nitrogen flow. The sample was heated to 950 °C with a heating rate of 5 °C min^−1^ and kept for 2 h. After naturally cooling down to room temperature, the obtained black powder was collected and directly used for further characterization and measurement. A similar method, as described above, was used for the preparation of NC and Co@NC with the precursor of ZIF-8 and ZIF-67, respectively.

### 2.4. Synthesis of Ru-pCo@NC

A total of 50 mg of the as-prepared pCo@NC nanoparticles was dispersed in 20 mL deionized water with ultrasonic stirring for 1 h to form a completely homogeneous suspension. Then, 0.048 mmol of RuCl_3_ was added to the above suspension. After reacting at 40 °C for 24 h in the shaker, the solid was collected, washed thoroughly with water and MeOH for several times, and finally dried in vacuum at 60 °C overnight. For comparison, a series of composites with 0.024, 0.096, and 0.144 mmol of RuCl_3_ were also obtained and denoted as Ru-pCo@NC-1, Ru-pCo@NC-2, and Ru-pCo@NC-3.

### 2.5. Characterization

The composition and structure of the samples were observed by X-ray diffraction (XRD) instrument (Cu Kα radiation) and Raman spectrometer. The surface morphologies and microscopic structure were characterized by scanning electron microscopy (SEM), high-resolution transmission electron microscopy (TEM), high-angle annular dark field-scanning transmission electron microscopy (HAADF-STEM), and energy dispersive X-ray (EDX) spectroscopy. N_2_ adsorption/desorption measurements were carried out for SSA and pore size distribution. Further composition and valence state information were obtained by X-ray photoelectron spectroscopy (XPS).

### 2.6. Electrochemical Measurements

The electrochemical measurements were conducted on a CHI 660E electrochemical workstation with a typical three-electrode setup in 1 M KOH electrolytes at about 25 °C. The catalyst coated on carbon fiber cloth was used as the working electrode, an Ag/AgCl electrode in saturated KCl as the reference electrode, and a graphite rod as the counter electrode. The working electrode was prepared as follows: 5.0 mg of the catalyst was dispersed in the mixture of distilled water (0.45 mL) and Nafion (5 wt%, 0.05 mL) and ultrasonicated for 1 h to form a uniform suspension. Then, 50 μL of the suspension was dropped onto a piece of clean carbon fiber cloth (0.5 cm^2^) and dried at room temperature.

The linear sweep voltammetry (LSV) curves were collected from −0.8 to −1.8 V (vs. Ag/AgCl) with a scan rate of 5 mV s^−1^. All potentials reported in this work were converted to the reversible hydrogen electrode (RHE) scale according to *E* (RHE) = *E* (Ag/AgCl) + 0.059pH + 0.197 (25 °C). In addition, All polarization curves were corrected by the *iR* contribution within the system following the equation of E (*iR* corrected) = E (RHE) − *iR*, where *i* is the current and *R* is the solution resistance measured by electrochemical impedance spectroscopy (EIS) measurements. EIS was examined under alternating-current voltage amplitude of 5 mV with a frequency from 0.01 to 10^6^ Hz. The electrochemical double-layer capacitance (C_dl_) was determined by the cyclic voltammograms (CVs) measured at different scan rates from 20 to 100 mV s^−1^ in the potential range of 0.1 to 0.3 V (vs. RHE). The stability tests were carried out through CV method with potential scanning between −0.2 to −0.04 V (vs. RHE) at 50 mV s^−1^ for 10,000 cycles and chronoamperometry at a constant overpotential of 43 mV (vs. RHE).

## 3. Results and Discussion

### 3.1. Structural Characterization

**Figure 1:**
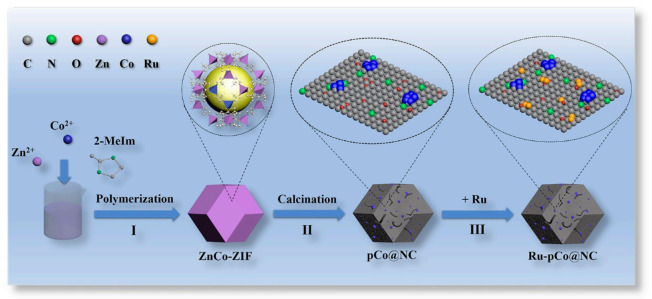
Schematic illustration of the Ru-pCo@NC preparation process.

The synthesis schematic diagram of Ru-pCo@NC is outlined in Figure 1. Briefly, the ZnCo-ZIF is firstly synthesized by the self-assembly of Zn^2+^ ions, Co^2+^ ions (the Zn/Co molar ratio = 7:3), and 2-methylimidazole (2-MeIm) ligands at room temperature. Then, the ZnCo-ZIF precursor is calcinated to form N-doped porous carbon with metallic Co NPs encapsulated and some nanotubes decorated (pCo@NC). Subsequently, low content Ru species (determined as 3.13 wt% by inductively coupled plasma-mass spectrometry (ICP-MS)) are loaded onto the carbon skeleton to obtain the Ru-pCo@NC.

**Figure 2:**
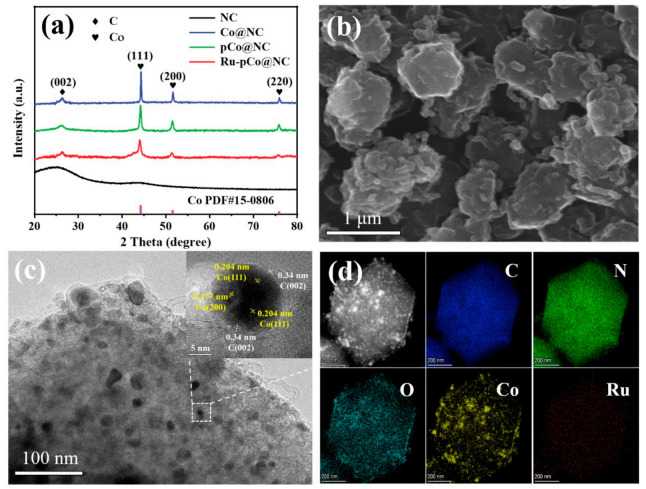
(**a**) XRD patterns of NC, Co@NC, pCo@NC, and Ru-pCo@NC. (**b**) SEM image, (**c**) TEM image and HRTEM image (insert), and (**d**) HAADF-STEM and corresponding EDX elemental mapping images of Ru-pCo@NC.

The XRD patterns of the as-synthesized ZIF-8, ZIF-67, and ZnCo-ZIF are presented in Figure S1a, which are well matched to that of the simulated ZIF-67 due to their similar crystal phase, indicating the ZIF structures are successfully synthesized. For the pattern of pure NC derived from the ZIF-8 (Figure 2a), the two bulging peaks at around 26° and 44° belong to the amorphous carbon. For those of Co@NC, pCo@NC, and Ru-pCo@NC, the diffraction peak found at 26.2° can be designated as the (002) facet of graphitic carbon. Obviously, the presence of Co can improve the graphitization, which is beneficial to the electrical conductivity. Another three prominent peaks at 44.2°, 51.5°, and 75.9° correspond to the (111), (200), and (220) facets of face-centered-cubic (*fcc*) Co (JCPDS card No. 15-0806), respectively [25]. Notably, no characteristic diffraction peak relevant to Ru species is marked in the XRD pattern of Ru-pCo@NC, most probably owing to their smaller size and low crystallinity [13], as discussed further below. Moreover, the XRD patterns of Ru-pCo@NC-1, Ru-pCo@NC-2, and Ru-pCo@NC-3 composites show almost the same, indicating that the increase in Ru amount has no effect on their crystal structure (Figure S1b).

The SEM is used for the morphology observation of the samples. As seen in Figure S2a–c, the as-synthesized ZIF-8, ZIF-67, and ZnCo-ZIF are all in distinct rhombic polyhedron shapes with relatively flat surfaces and average size of ~700–800 nm. The obtained NC inherits the overall polyhedron structure of the ZIF-8 precursor (Figure S2d). For Co@NC, plenty of larger-sized Co particles are distributed on the surfaces due to severe agglomeration under the high-temperature pyrolysis condition (Figure S2e). In contrast, there are no apparent large Co NPs formed on the surfaces of pCo@NC, because the introduction of Zn act as a spacer between the Co sites in the ZnCo-ZIF precursor, and Zn species will evaporate during the carbonization process [26], thus preventing the further aggregation of Co. Moreover, the surfaces of pCo@NC are decorated with some curved carbon nanotubes (CNTs) (Figure S2f), which should be converted from the organic skeleton by the catalysis of Co NPs [27]. The morphological features of Ru-pCo@NC are revealed in Figure 2b, and there is no noticeable change compared with that of pCo@NC, indicating that the addition of Ru nearly has no effect on the morphology of pCo@NC support. In the TEM image of Ru-pCo@NC (Figure 2c), many metal nanoparticles with a diameter range of 5~40 nm are homogeneously embedded in the carbon matrix. Moreover, for the HRTEM image of Ru-pCo@NC (insert of Figure 2c), the distinct lattice spaces of the particle are measured to be 0.204 and 0.177 nm, corresponding to Co (111) and (200) planes of the cubic phase, respectively. Abundant graphitic carbon layers can be observed around the Co NPs and show the interplanar spacing of the carbon (002) crystal plane (0.34 nm). The outer graphite layers could availably prevent the accumulation of Co NPs, thus improving the activity and durability of catalyst in the electrochemical test. Additionally, the direct interfacial interaction between the Co NPs and outer carbon layers would enhance electrical conductivity and favor electron transfer [28]. Moreover, no definite space lattice of Ru species is found, which coincides with the result of XRD analysis and further proves the low crystallinity of Ru. In Figure 2d, the HAADF-STEM and corresponding EDX elemental mapping spectroscopy of Ru-pCo@NC corroborate the existence of C, N, O, Co, and Ru in the composite, from which it can be seen that Ru species are homogeneously dispersed on the support without obvious aggregation. Therefore, it is referred that Ru exists in the form of smaller nanoclusters.

**Figure 3:**
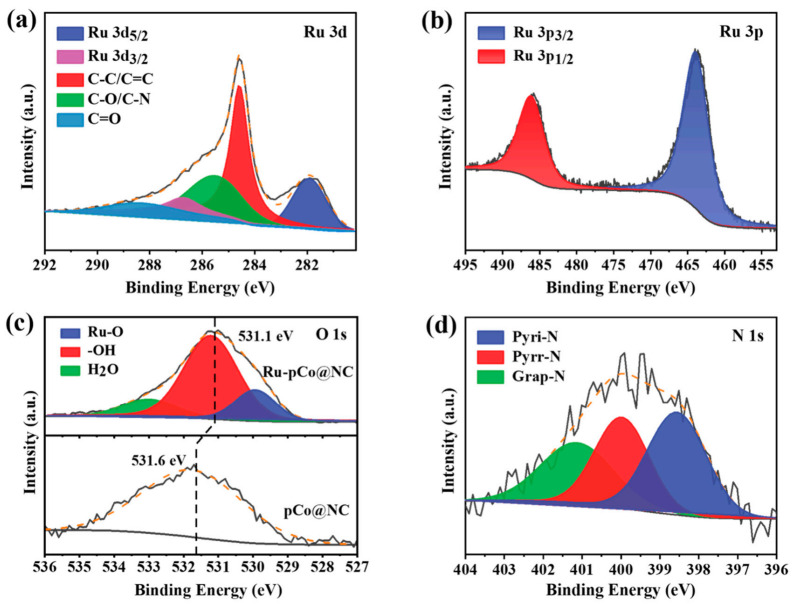
High-resolution XPS spectra of (**a**) Ru 3d (partially overlaps with C 1s) and (**b**) Ru 3p of Ru-pCo@NC, (**c**) O 1s of Ru-pCo@NC and Co@NC, and (**d**) N 1s of Ru-pCo@NC.

The elemental composition and electron states of Ru-pCo@NC are detected by XPS. The survey spectra of pCo@NC and Ru-pCo@NC in Figure S3a disclose C, N, O, and Co elements in both samples and additional Ru element in Ru-pCo@NC. As can been seen in the high-resolution XPS spectrum of Zn 2p in Ru-pCo@NC (Figure S3b), the Zn signal is hardly detected, and the Zn content is measured to be only 0.12%, confirming the almost evaporation of Zn species during the high-temperature calcination process. In Figure 3a, the high-resolution spectrum of Ru 3d in Ru-pCo@NC, which partially overlaps with C 1s, can be deconvoluted into Ru 3d_5/2_ (281.8 eV) and Ru 3d_3/2_ (286.4 eV) of zero-valence Ru (Ru^0^) [29], along with C–C/C=C (284.6 eV), C–O/C–N (285.5 eV), and C=O (288.4 eV) [30]. It is known that the Ru 3p_3/2_ and Ru 3p_1/2_ peaks of Ru^0^ generally appear at 462.5 and 484.9 eV [31]. From the Ru 3p spectrum of Ru-pCo@NC (Figure 3b), the Ru 3p_3/2_ and Ru 3p_1/2_ peaks have higher binding energies and locate at 463.1 and 485.6 eV, respectively. Significantly, the increase in binding energy means the electron deficiency, implying that there is an electronic effect between Ru and the surrounding species. On the contrary, a negative shift in binding energy is detected in the O 1s spectrum of Ru-pCo@NC compared with that of pCo@NC (Figure 3c). In addition, the oxygen species are identified as the Ru–O bond (529.8 eV), –OH (531.3 eV), and physically or chemically bonded H_2_O (533.2 eV) on the carbon surface [32] from the O 1s spectrum of Ru-pCo@NC. Therefore, the increased binding energy of Ru and the decreased binding energy of O can arise from the electronic effect between Ru and O species on the support, thus resulting in the electron transfer from Ru to O [33]. As proved by previous studies, Ru has a moderated hydrogen binding energy that was close to that of Pt [9,34]. In our case, the interaction between Ru and O should lead to electron redistribution within the composite, thus facilitating the charge transfer and reaction kinetics to produce Ru–H* for the HER. The high-resolution N 1s curve of Ru-pCo@NC is divided into the pyridinic N (398.6 eV), pyrrolic N (400.0 eV), and graphitic N (401.1 eV) species (Figure 3d) [35]. Some studies have explained that these N doping can induce changes in the electronic structure of the carbon matrix, thereby also leading to a certain degree of promotion in electrochemical performance of carbon materials [36,37]. The deconvoluted spectrum of Co 2p reveals that metallic Co (Co^0^) and Co^2+^ species coexist in Ru-pCo@NC (Figure S3c) [11]. The presence of cobalt oxides could be attributed to the exposure of samples in air, which can be explained because the XPS analysis concentrates primarily on the surface chemical elements of a sample (about ~3–5 nm) [38].

**Figure 4:**
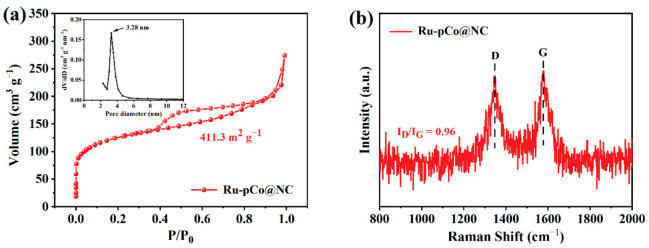
(**a**) N_2_ adsorption/desorption isotherms and pore size distribution (insert) and (**b**) Raman spectrum of Ru-pCo@NC.

The pore structure and SSA of the samples are analyzed by the N_2_ adsorption/desorption measurement. Co@NC and pCo@NC both show the type-IV isotherms (Figure S4a), demonstrating the existence of mesopores in the samples. When the relative pressure is low (P/P_0_ < 0.1), the adsorption capacity of pCo@NC for N_2_ is seen as higher than that of Co@NC, signifying more abundant pores in pCo@NC. The SSAs of Co@NC and pCo@NC determined by the Brunauer–Emmett–Teller (BET) method are 132.5 and 428.2 m^2^ g^−1^, respectively (see detailed structure information in Table S2). Obviously, the bimetallic ZnCo-ZIF as a precursor can provide significantly increased porosity and SSA of support owing to the evaporation of Zn under the high-temperature carbonization condition. The insert of Figure S4a is the pore size distributions of Co@NC and pCo@NC. More importantly, Ru-pCo@NC retains the high SSA of 411.3 m^2^ g^−1^ and mesopore characteristic of pCo@NC (Figure 4a). In addition, the pore size distribution of Ru-pCo@NC (insert of Figure 4a) displays a sharp peak centered at ~3.28 nm. As we all know, a large SSA of electrocatalyst is important to the exposure and accessibility of active species, and mesopores are conducive to the mass transport in the electrocatalytic process and accelerate the reaction rate. Further, the carbon components of these samples are probed by Raman spectroscopy. The two pronounced vibrational peaks at 1350 and 1580 cm^–1^ are characteristics of the D and G bands for carbon materials (Figure S4b), which can be used as indicators of the defective component and graphitic component, respectively [39]. The intensity ratio of D band and G band (*I_D_*/*I_G_*) is measured to be 0.94 for pCo@NC, higher than that of Co@NC (*I_D_*/*I_G_* = 0.81), implying that pCo@NC is less graphitized than Co@NC due to the formation of more pores and defects, and these defect sites have proven helpful for electrochemical catalysis [40]. The *I_D_*/*I_G_* value of Ru-pCo@NC is calculated to be 0.96 (Figure 4b), demonstrating that the incorporation of Ru into pCo@NC still retains the advantageous carbon structure of the support.

### 3.2. Electrocatalytic HER Characterization

The HER catalytic behaviors of the as-obtained catalysts are examined in 1 M KOH electrolyte. Figure S5 depicts the *iR*-corrected LSV curves of the Ru-pCo@NC composites with different Ru amount. Accordingly, the *η*_10_ values are measured to be 55, 30, 29, and 29 mV for Ru-pCo@NC-1, Ru-pCo@NC, Ru-pCo@NC-2, and Ru-pCo@NC-3, respectively. It is noteworthy that the catalytic activity initially improves and then remains stable with Ru amount increasing, which is possibly because the Ru precursor is excessive. In consideration of cost, the Ru-pCo@NC is selected as the optimum catalyst for further investigation, as described below.

**Figure 5:**
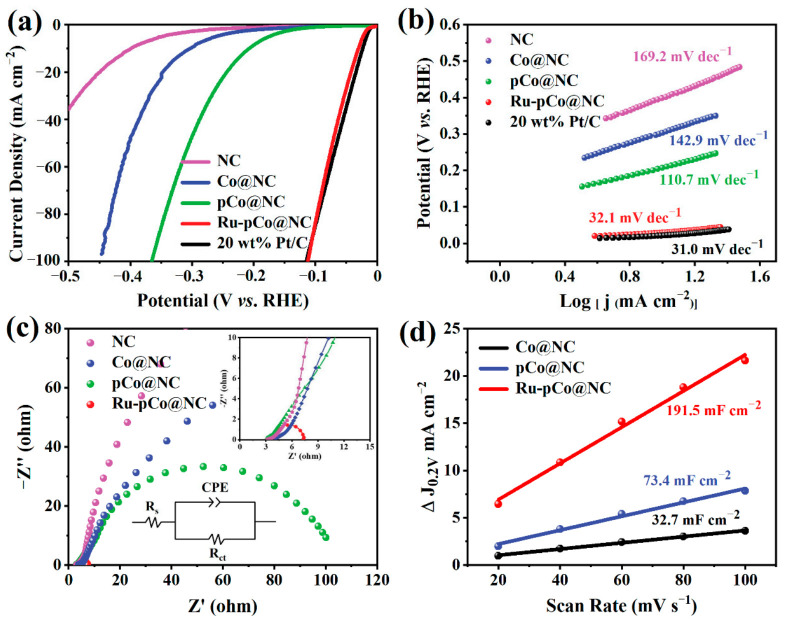
(**a**) *iR*-corrected LSV curves and (**b**) Tafel plots of NC, Co@NC, pCo@NC, Ru-pCo@NC, and 20 wt% Pt/C in 1 M KOH. (**c**) Nyquist plots of NC, Co@NC, pCo@NC, and Ru-pCo@NC (the magnified curves and equivalent circuit are presented in the insert of c). (**d**) Relationship curves of capacitive current density (ΔJ = (J_a_ − J_c_)/2) at 0.2 V versus scan rate for the determination of C_dl_s of Co@NC, pCo@NC, and Ru-pCo@NC.

The LSV curves of NC, Co@NC, pCo@NC, Ru-pCo@NC, and commercial 20 wt% Pt/C are presented in Figure 5a. As observed, the HER catalytic behavior of Co@NC is superior to NC, and pCo@NC delivers an obvious performance improvement (*η*_10_ = 207 mV), which proves the combined effect of graphitization, SSA, and porosity on HER catalytic activity. Furthermore, great activity promotion is observed for Ru-pCo@NC (*η*_10_ = 30 mV), almost catching up with the catalytic performance of Pt/C (*η*_10_ = 21 mV), implying that the Ru nanoclusters serve as the main active sites. In order to compare the HER kinetics rates on the above electrocatalysts, Tafel slopes are taken from the corresponding LSV curves applying the Tafel equation [1]. In Figure 5b, Ru-pCo@NC delivers a low Tafel slope of 32.1 mV dec^−1^ according to the Tafel plot depicted, which is approaching to that of Pt/C (31.0 mV dec^−1^), and much smaller than those of NC (169.2 mV dec^−1^), Co@NC (142.9 mV dec^−1^), and pCo@NC (110.7 mV dec^−1^), implying that a faster HER reaction kinetics is achieved in Ru-pCo@NC, which can be attributed to the interaction between Ru and O, as revealed by the above XPS analysis. Such a superior Tafel slope demonstrates that the HER proceeding on Ru-pCo@NC electrocatalyst follows the very efficient Volmer–Tafel mechanism, with the formation of molecular hydrogen between two adjacently adsorbed H atom (H* + H* → H_2_) as the rate-determining step (RDS) [41]. Subsequently, the charge transfer resistance (R_ct_) of the electrocatalyst is evaluated by EIS. The Nyquist plots of NC, Co@NC, pCo@NC, and Ru-pCo@NC are compared in Figure 5c. As depicted, the R_ct_ values follow the order of Ru-pCo@NC (4.3 Ω) < pCo@NC (102.2 Ω) < Co@NC (307.6 Ω) < NC, confirming that an extremely fast charge transfer is realized in Ru-pCo@NC, thus speeding up the reaction kinetics. Moreover, the Tafel slopes and R_ct_ values of Ru-pCo@NC-1, Ru-pCo@NC-2, and Ru-pCo@NC-3 are also compared in Figure S6a and Figure S6b, respectively. It follows the same trend of their catalytic behavior, that is, first decreasing gradually and then remaining stable. The CV curves of Co@NC, pCo@NC, and Ru-pCo@NC recorded at increased scan rates (20 to 100 mV s^−1^) are depicted in Figure S7a–c. Accordingly, the C_dl_ of Ru-pCo@NC is estimated to be 95.7 mF cm^−2^, much higher than that of Co@NC (16.4 mF cm^−2^) and pCo@NC (36.7 mF cm^−2^), meaning more exposed electrochemically active sites in Ru-pCo@NC (Figure 5d). This is as a result of its high SSA, abundant pores, and well-dispersed Ru active sites.

**Figure 6:**
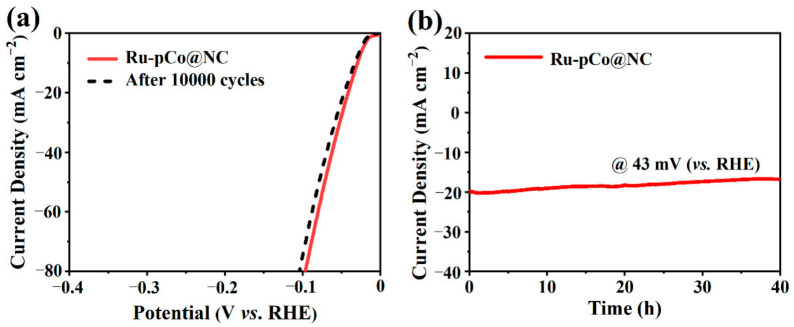
(**a**) Comparison of LSV curves for Ru-pCo@NC before and after 10000 CV cycles in 1 M KOH and (**b**) the chronoamperometric curve of Ru-pCo@NC at a constant overpotential of 43 mV (vs. RHE).

Moreover, another crucial target for electrocatalyst in practical applications is its stability. The cycling stability of Ru-pCo@NC is evaluated by 10,000 CV cycles in 1 M KOH. Significantly, the catalytic ability of Ru-pCo@NC shows negligible attenuation with the *η*_10_ increasing by only 5 mV (Figure 6a). Moreover, the long-term durability of Ru-pCo@NC is measured by chronoamperometry, and the obtained current-time (*i-t*) curve (Figure 6b) reveals that the catalytic activity of Ru-pCo@NC could be maintained for at least 40 h under the alkaline condition. After durability testing, the polyhedral morphology of Ru-pCo@NC is well preserved, and Ru is still dispersed on the carbon support uniformly (Figure S8). Therefore, these results firmly manifest that the robust stability of the Ru active species and the porous carbon support.

The electrochemical performance of Ru-pCo@NC catalyst in an acid electrolyte (0.5 M H_2_SO_4_) is also described in Figure S9. The *η*_10_ and Tafel slope of Ru-pCo@NC are 64 mV and 69.5 mV dec^−1^, respectively (Figure S9a,b). Such performance shows slightly inferior to those of 20 wt% Pt/C (*η*_10_ = 35 mV, 32.4 mV dec^−1^) but compare favorably with those of Co@NC (*η*_10_ = 451 mV, 239.7 mV dec^−1^) and pCo@NC (*η*_10_ = 258 mV, 114.7 mV dec^−1^), as well as most reported HER catalysts under acid condition (Table S3). The R_ct_ of Ru-pCo@NC determined by the Nyquist plot is 8.2 Ω (Figure S9c), representing its fast electron transfer for HER in acid solution. Subsequently, the long-term stability of Ru-pCo@NC is also tested. As observed in Figure S9d, the activity of Ru-pCo@NC almost remains stable over testing for 15,000 s, while that of 20 wt% Pt/C shows a noticeable decay in 8000 s, further confirming that the satisfactory stability of Ru-pCo@NC in acid solution.

## 4. Conclusions

In summary, a high-performance HER electrocatalyst by introducing low-content Ru nanoclusters onto the Co/N-codoped porous carbon derived from a bimetallic ZIF is synthesized. Thanks to the large SSA and high porosity of carbon support, uniformly dispersed Ru active sites, and rapid reaction kinetics of the interaction between Ru and O, the Ru-pCo@NC catalyst exhibits outstanding catalytic activity in 1 M KOH electrolyte with a low overpotential of 30 mV at the current density of 10 mA cm^−2^, rivaling that of commercial Pt/C. In addition, Ru-pCo@NC also displays a desirable stability for long-time running. It is demonstrated that the small Ru nanoclusters as the main active sites in Ru-pCo@NC play a critical role in accelerating the HER kinetics. Due to the great flexibility in the composition and structure of MOFs, this study provides a potential idea for preparing highly effective and stable HER catalysts with a favorable support structure and uniformly dispersed active sites, which is of great significance for the economic efficiency in practical applications.

## Data Availability

The data presented in this study are available in this article and its Supplementary Materials.

## Supplementary Materials

The following are available online at https://www.mdpi.com/article/10.3390/nano11051228/s1, Figure S1: XRD patterns of (a) as-synthesized ZIE-8, ZIF-67, ZnCo-ZIF, and simulated ZIF-67; and (b) Ru-pCo@NC-1, Ru-pCo@NC, Ru-pCo@NC-2, and Ru-pCo@NC-3. Figure S2: SEM images of (a) ZIF-8, (b) ZIF-67, (c) ZnCo-ZIF; (d) NC; (e) Co@NC; and (f) pCo@NC. Figure S3: XPS spectra of Ru-pCo@NC: (a) survey spectrum; (b) Zn 2p; and (c) Co 2p. Figure S4: N_2_ adsorption/desorption isotherms and (b) Raman spectra of Co@NC and pCo@NC. Figure S5: LSV curves of of Ru-pCo@NC-1, Ru-pCo@NC, Ru-pCo@NC-2, and Ru-pCo@NC-3 in 1 M KOH. Figure S6: (a) Tafel plots and (b) Nyquist plots of Ru-pCo@NC-1, Ru-pCo@NC, Ru-pCo@NC-2, and Ru-pCo@NC-3 in 1 M KOH. Figure S7: CV curves of (a) Co@NC; (b) pCo@NC; and (c) Ru-pCo@NC. Figure S8: (a) TEM image, (b) HRTEM image, and (c) HAADF-STEM and corresponding EDX elemental mapping images of Ru-pCo@NC used after chronoamperometric measurements. Figure S9: The electrochemical performance of the as-prepared catalysts in 0.5 M H_2_SO_4_: (a) LSV curves; (b) Tafel plots; (c) Nyquist plot; and (d) chronoamperometric curve. Table S1: Comparison of Ru-based electrocatalysts towards HER in alkaline electrolyte. Table S2: Comparison of structure features for Co@NC and pCo@NC. Table S3: Comparison of the HER performance of Ru-pCo@NC with reported electrocatalysts in acidic electrolyte.
