# Supplementary material for: Bimetallic ZIF-Derived Co/N-Codoped Porous Carbon Supported Ruthenium Catalysts for Highly Efficient Hydrogen Evolution Reaction
Source: Nanomaterials (Basel). 2021 May 6;11(5):1228. doi: 10.3390/nano11051228 (PMC8148513; doi:10.3390/nano11051228)
Supplement: Supplementary file 1 [file nanomaterials-11-01228-s001.zip › nanomaterials-1199248-supplementary.pdf]

Supporting Information

Bimetallic ZIF-Derived Co/N-Codoped Porous Carbon-Supported Ruthenium Catalysts for Highly Efficient Hydrogen Evolution Reaction

Hui Qi †, Xinglong Guan †, Guangyu Lei, Mengyao Zhao, Hongwei He, Kai Li, Guoliang Zhang, Fengbao Zhang, Xiaobin Fan, Wenchao Peng, and Yang Li *

Lab of Advanced Nano-Structure and Transfer Process, Department of Chemical Engineering, Tianjin University, Tianjin 300354, China; qihui1101@tju.edu.cn (H.Q.); tjugxl@tju.edu.cn; (X.G); guangyulei@tju.edu.cn (G.L.); Zhaomy@tju.edu.cn (M.Z.); hehongwei@tju.edu.cn (H.H); tju-likai@tju.edu.cn (K.L.); zhangguoliang@tju.edu.cn (G.Z.); fbzhang@tju.edu.cn (F.Z.); xiaobinfan@tju.edu.cn (X.F.); wenchao.peng@tju.edu.cn (W.P.)

* Correspondence: liyang1895@tju.edu.cn; Tel.: +86-22-2789-0090

† The authors contribute equal to this study.

Citation: Qi, H.; Guan, X.; Lei, G.; Zhao, M.; He, H.; Li, K.; Zhang, G.; Zhang, F.; Fan, X.; Peng, W.; et al. Bimetallic ZIF-Derived Co/N-Codoped Porous Carbon-Supported Ruthenium Catalysts for Highly Efficient Hydrogen Evolution Reaction. *Nanomaterials* **2021**, *11*, 1228. https://doi.org/10.3390/nano11051228

Academic Editor: Akinobu Yamaguchi

Received: 11 April 2021
Accepted: 28 April 2021
Published: 6 May 2021

Publisher's Note: MDPI stays neutral with regard to jurisdictional claims in published maps and institutional affiliations.

Copyright: © 2021 by the authors. Submitted for possible open access publication under the terms and conditions of the Creative Commons Attribution (CC BY) license (http://creativecommons.org/licenses/by/4.0/).

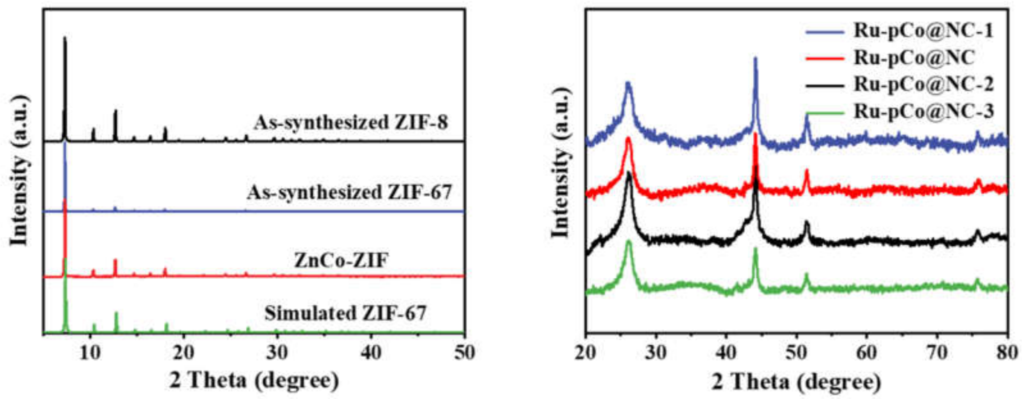

Figure S1. (a) XRD patterns of as-synthesized ZIE-8, ZIF-67, ZnCo-ZIF, and simulated ZIF-67; (b) XRD patterns of Ru-pCo@NC-1, Ru-pCo@NC, Ru-pCo@NC-2, and Ru-pCo@NC-3.

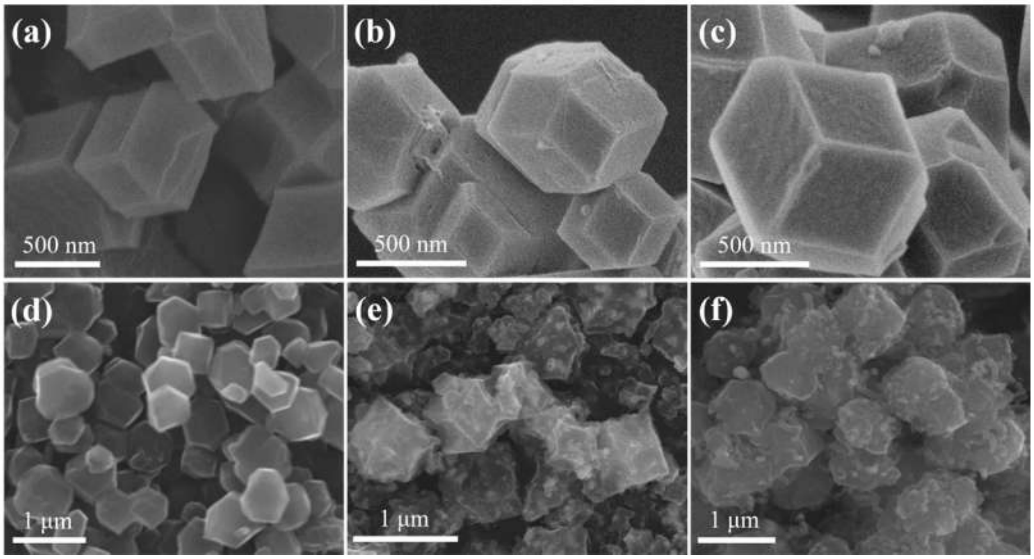

Figure S2. (a-c) SEM images of as-synthesized ZIF-8, ZIF-67 and ZnCo-ZIF with Zn/Co molar ratio of 7:3, respectively; (d-f) SEM images of their pyrolysis products NC, Co@NC and pCo@NC, respectively.

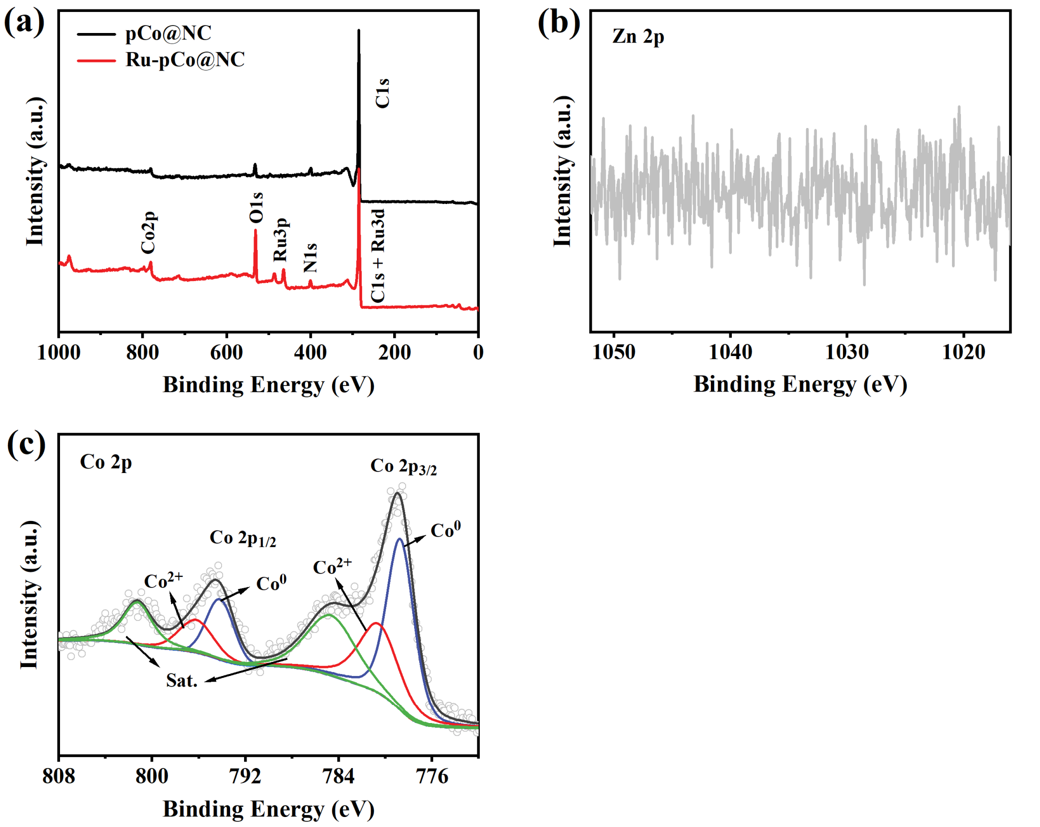

Figure S3. (a) XPS survey spectra of pCo@NC and Ru-pCo@NC, (b) high-resolution Zn 2p spectrum and (c) high-resolution Co 2p spectrum of Ru-pCo@NC.

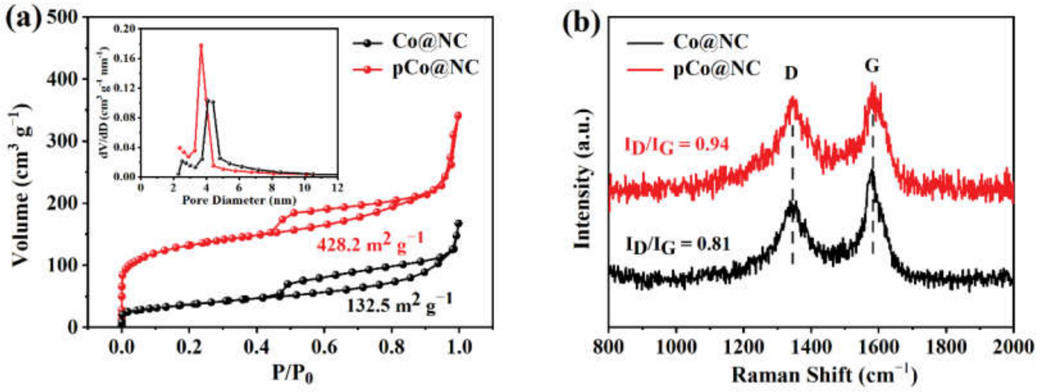

Figure S4. (a) N_2 adsorption/desorption isotherms and pore size distributions (insert) and (b) Raman spectra of Co@NC and pCo@NC.

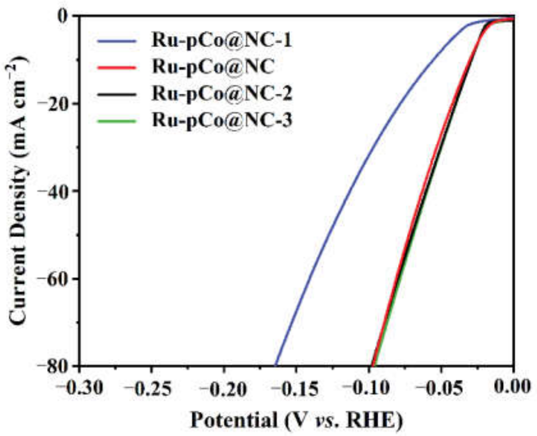

Figure S5. LSV curves of of Ru-pCo@NC-1, Ru-pCo@NC, Ru-pCo@NC-2, and Ru-pCo@NC-3 in 1 M KOH.

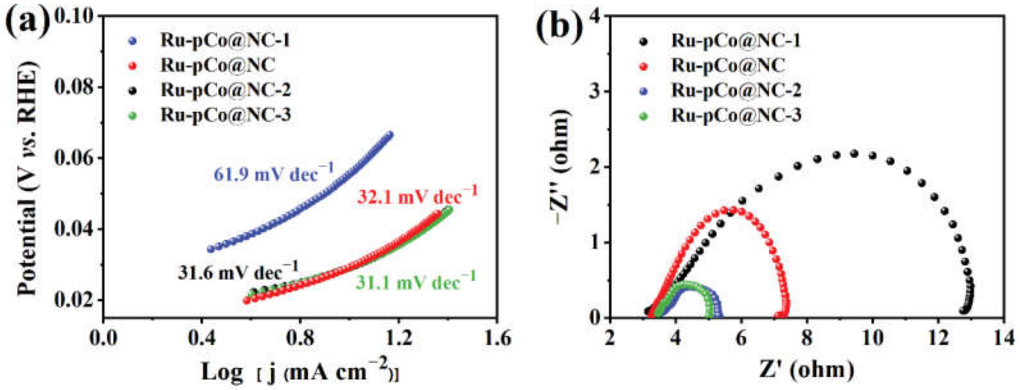

Figure S6. (a) Tafel plots and (b) Nyquist plots of Ru-pCo@NC-1, Ru-pCo@NC, Ru-pCo@NC-2, and Ru-pCo@NC-3 in 1 M KOH.

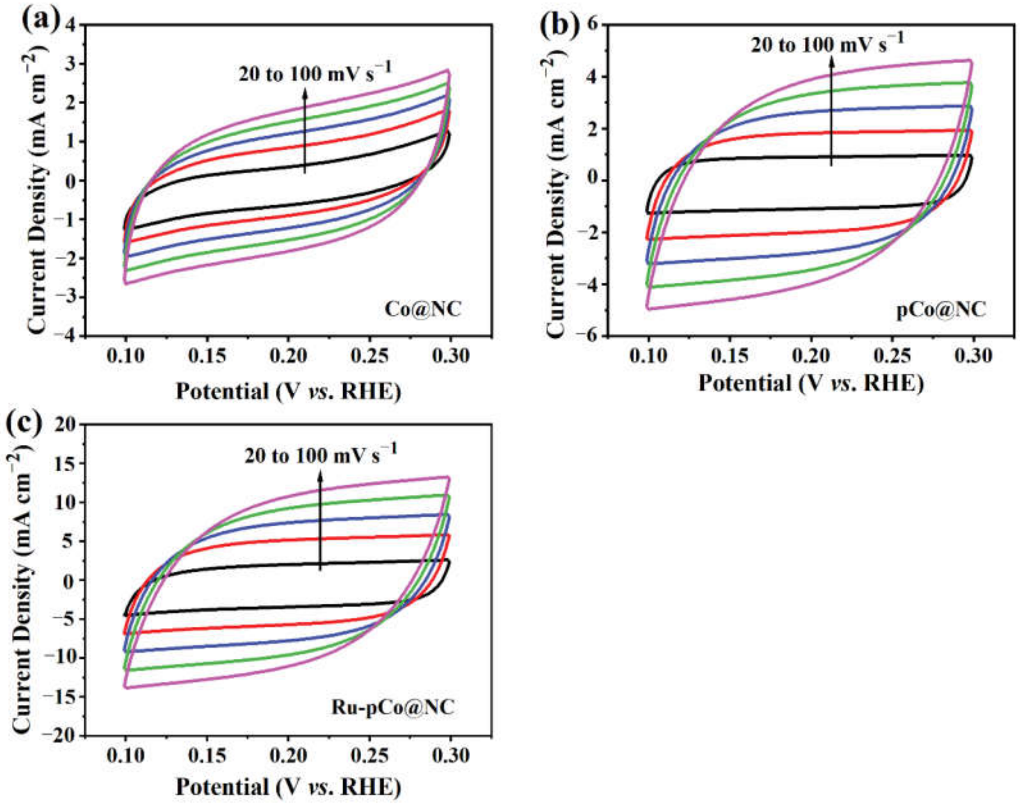

Figure S7. Cyclic voltammetry (CV) curves of (a) Co@NC, (b) pCo@NC and (c) Ru-pCo@NC from 0.1 to 0.3 V (*vs*. RHE) with scan rate from 20 to 100 mV s^{-1} in 1 M KOH, respectively.

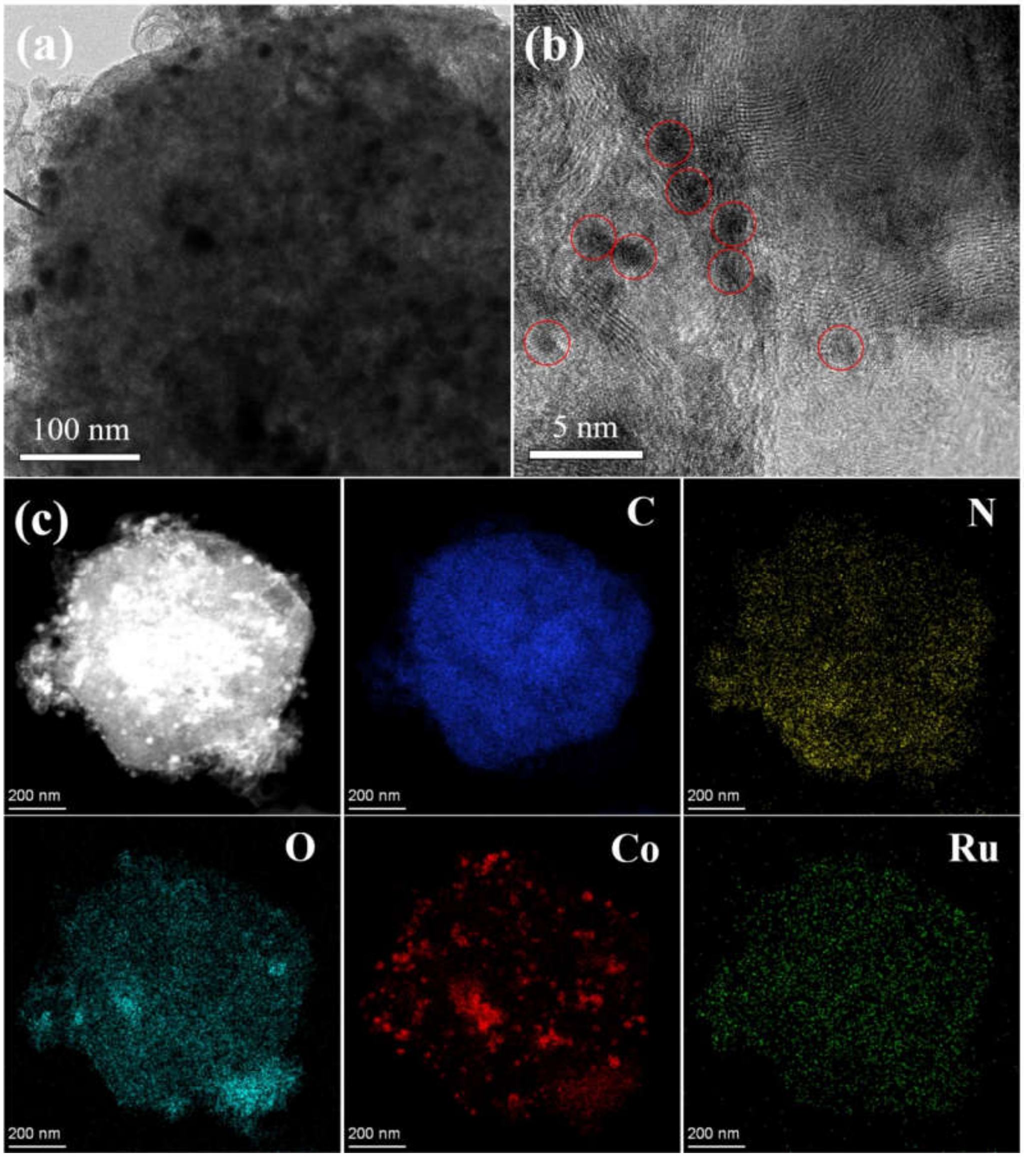

Figure S8. (a) TEM image, (b) HRTEM image, and (c) HAADF-STEM and corresponding EDX elemental mapping images of Ru-pCo@NC used after chronoamperometric measurements.

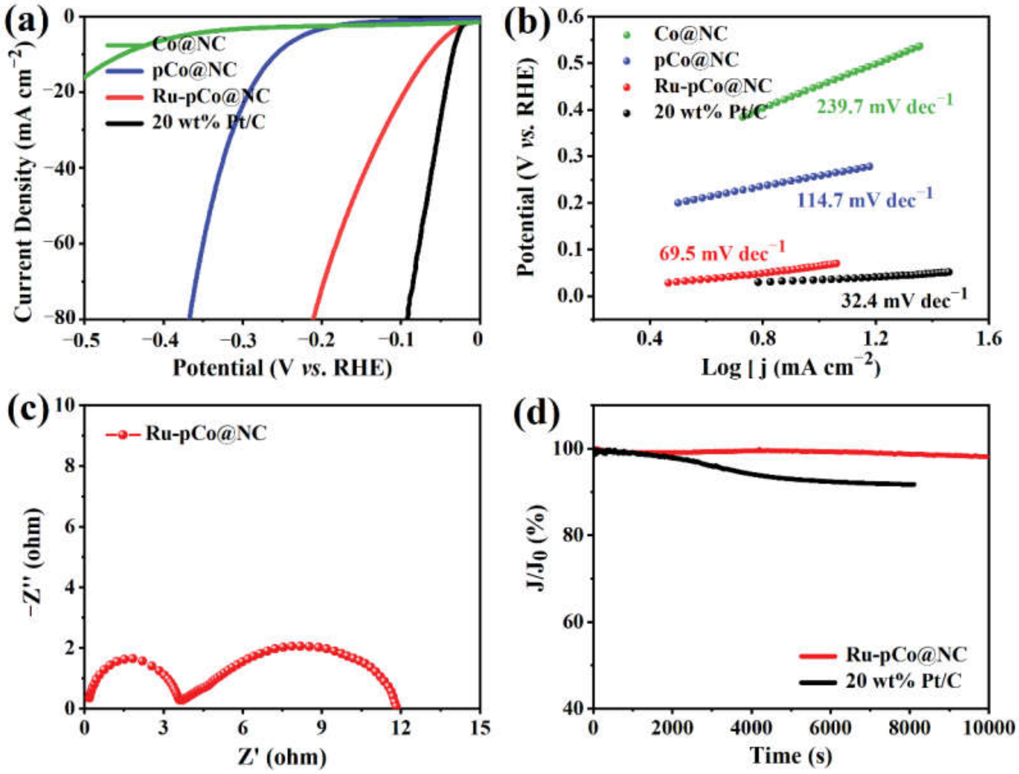

Figure S9. The electrochemical performance of the as-prepared catalysts in 0.5 M H_2SO_4. (a) *iR*-corrected LSV curves and (b) corresponding Tafel plots of Co@NC, pCo@NC, Ru-pCo@NC and 20 wt % Pt/C; (c) Nyquist plot of Ru-pCo@NC with the frequency range of 0.01 Hz to 10^6 Hz at an amplitude of 5 mV; (d) Comparison of long-term durability for Ru-pCo@NC and 20 wt% Pt/C by chronoamperometry test.

Table S1. Comparison of Ru-based electrocatalysts towards HER in alkaline electrolyte.

Catalysts	Electrolyte	Overpotential @ 10 mA cm^{-2} (mV)	Tafel slope (mV dec^{-1})	Reference
Ru-pCo@NC	1 M KOH	30	32.1	This work
RuO_2/F-grephene	1 M KOH	49	31	[1]
RuO_2@C	1 M KOH	35	46	[2]
Ru/C_3N_4/C	0.1 M KOH	79	~71	[3]
Ru NP/C	1 M KOH	52	33	[4]
RuP_2@NPC	1 M KOH	52	69	[5]
Ru_2Ni_2 SNs/C	1 M KOH	40	23.7	[6]
Ru@Co-NC	1 M KOH	23	58.1	[7]
Ru/Cu-doped RuO_2)	1 M KOH	41	51	[8]
1D-RuO_2-CN_x	0.5 M KOH	95	70	[9]
Ni@Ni_2P-Ru	1 M KOH	31	41	[10]

Table S2. Comparison of structure features for Co@NC and pCo@NC.

Samples	BET Surface Area (m^2/g)	Total Pore Volume (cm^3/g)	Average Pore Size (nm)
Co@NC	132.5	0.259	3.91
pCo@NC	428.2	0.517	2.41
Ru-pCo@NC	411.3	0.459	2.23

Table S3. Comparison of the HER performance of Ru-pCo@NC with reported electrocatalysts in acidic electrolyte.

Catalysts	Electrolyte	Overpotential @ 10 mA cm^{-2} (mV)	Tafel slope (mV dec^{-1})	Reference
Ru-pCo@NC	0.5 M H_2SO_4	64	69.5	This work
Ru@Co-NC	0.5 M H_2SO_4	110	102.5	[7]
1D-RuO_2-CN_x	0.5 M H_2SO_4	93	40	[9]
Ru/C_3N_4/C	0.5 M H_2SO_4	~75	~50	[3]
Ru-SA/$Ti_3C_2T_x$	0.1 M $HClO_4$	70	27.7	[11]
Mo_xC-IOL	0.5 M H_2SO_4	117	60	[12]
CoP/CC	0.5 M H_2SO_4	67	51	[13]
$NiCo_2P_x$/CF	0.5 M H_2SO_4	104	59.6	[14]
Fe-doped CoP/Ti	0.5 M H_2SO_4	78	75	[15]

Supplymental Reference

1. Fan, Z., Liao, F., Shi, H., Liu, Y., Shao, M., Kang, Z., Highly efficient water splitting over a RuO2/F-doped graphene electrocatalyst with ultra-low ruthenium content, Inorg. Chem. Front. 2020, 7, 2188-2194.
2. Park, H.-S., Yang, J., Cho, M.K., Lee, Y., Cho, S., Yim, S.-D., Kim, B.-S., Jang, J.H., Song, H.-K., RuO2 nanocluster as a 4-in-1 electrocatalyst for hydrogen and oxygen electrochemistry, Nano Energy 2019, 55, 49-58.
3. Zheng, Y., Jiao, Y., Zhu, Y., Li, L.H., Han, Y., Chen, Y., Jaroniec, M., Qiao, S.-Z., High Electrocatalytic Hydrogen Evolution Activity of an Anomalous Ruthenium Catalyst, J. Am. Chem. Soc. 2016, 138, 16174-16181.
4. Wang, Q., Ming, M., Niu, S., Zhang, Y., Fan, G., Hu, J.-S., Scalable Solid-State Synthesis of Highly Dispersed Uncapped Metal (Rh, Ru, Ir) Nanoparticles for Efficient Hydrogen Evolution, Adv. Energy Mater. 2018, 8.
5. Pu, Z., Amiinu, I.S., Kou, Z., Li, W., Mu, S., RuP2-Based Catalysts with Platinum-like Activity and Higher Durability for the Hydrogen Evolution Reaction at All pH Values, Angew. Chemie Int. Ed. 2017, 56, 11559-11564.
6. Ding, J., Shao, Q., Feng, Y., Huang, X., Ruthenium-nickel sandwiched nanoplates for efficient water splitting electrocatalysis, Nano Energy 2018, 47, 1-7.
7. Gao, H.W., Zang, J.B., Liu, X.X., Wang, Y.H., Tian, P.F., Zhou, S.Y., Song, S.W., Chen, P.P., Li, W., Ruthenium and cobalt bimetal encapsulated in nitrogen-doped carbon material derived of ZIF-67 as enhanced hydrogen evolution electrocatalyst, Appl. Surf. Sci. 2019, 494, 101-110.
8. Yang, K., Xu, P., Lin, Z., Yang, Y., Jiang, P., Wang, C., Liu, S., Gong, S., Hu, L., Chen, Q., Ultrasmall Ru/Cu-doped RuO2 Complex Embedded in Amorphous Carbon Skeleton as Highly Active Bifunctional Electrocatalysts for Overall Water Splitting, Small 2018, 14.
9. Bhowmik, T., Kundu, M.K., Barman, S., Growth of One-Dimensional RuO2 Nanowires on g-Carbon Nitride: An Active and Stable Bifunctional Electrocatalyst for Hydrogen and Oxygen Evolution Reactions at All pH Values, ACS Appl. Mater. Interfaces 2016, 8, 28678-28688.
10. Liu, Y., Liu, S., Wang, Y., Zhang, Q., Gu, L., Zhao, S., Xu, D., Li, Y., Bao, J., Dai, Z., Ru Modulation Effects in the Synthesis of Unique Rod-like Ni@Ni2P-Ru Heterostructures and Their Remarkable Electrocatalytic Hydrogen Evolution Performance, J. Am. Chem. Soc. 2018, 140, 2731-2734.
11. Peng, X., Zhao, S., Mi, Y., Han, L., Liu, X., Qi, D., Sun, J., Liu, Y., Bao, H., Zhuo, L., Xin, H.L., Luo, J., Sun, X., Trifunctional Single-Atomic Ru Sites Enable Efficient Overall Water Splitting and Oxygen Reduction in Acidic Media, Small 2020, 16.
12. Li, F., Zhao, X., Mahmood, J., Okyay, M.S., Jung, S.-M., Ahmad, I., Kim, S.-J., Han, G.-F., Park, N., Baek, J.-B., Macroporous Inverse Opal-like MoXC with Incorporated Mo Vacancies for Significantly Enhanced Hydrogen Evolution, Acs Nano 2017, 11, 7527-7533.
13. Tian, J., Liu, Q., Asiri, A.M., Sun, X., Self-Supported Nanoporous Cobalt Phosphide Nanowire Arrays: An Efficient 3D Hydrogen-Evolving Cathode over the Wide Range of pH 0-14, J. Am. Chem. Soc. 2014, 136, 7587-7590.
14. Zhang, R., Wang, X., Yu, S., Wen, T., Zhu, X., Yang, F., Sun, X., Wang, X., Hu, W., Ternary

NiCo2Px Nanowires as pH-Universal Electrocatalysts for Highly Efficient Hydrogen Evolution Reaction, Adv. Mater. 2017, 29.

15. Tang, C., Zhang, R., Lu, W., He, L., Jiang, X., Asiri, A.M., Sun, X., Fe-Doped CoP Nanoarray: A Monolithic Multifunctional Catalyst for Highly Efficient Hydrogen Generation, Adv. Mater. 2017, 29.
